# Clinical Profile of Eprosartan: A Different Angiotensin II Receptor Blocker

**DOI:** 10.2174/187152508785909500

**Published:** 2008-10

**Authors:** P. J Blankestijn, H Rupp

**Affiliations:** 1 Department of Nephrology, University Medical Center, Utrecht, The Netherlands; 2 Philipps University of Marburg, Department of Internal Medicine and Cardiology, Molecular Cardiology Laboratory, Marburg, Germany

**Keywords:** Eprosartan, hypertension, sympathetic nervous system, renin angiotensin system.

## Abstract

**Rationale.** The goal of antihypertensive treatment is to reduce risk of cardiovascular morbidity and mortality. Apart from blood pressure lowering *per se*, also reducing the activities of the renin-angiotensin system and sympathetic nervous system appears to be important. Angiotensin II receptor blocker drugs (ARBs) have provided a useful class of anti-hypertensive drugs. Eprosartan is a relatively new ARB which is chemically distinct (non-biphenyl, non-tetrazole) from all other ARBs (biphenyl tetrazoles). An analysis has been made on available experimental and clinical data on eprosartan which not only is an effective and well tolerated antihypertensive agent, but also lowers the activities of the renin-angiotensin system and sympathetic nervous system. Experimental and pharmacokinetic studies on eprosartan have shown differences with the other ARBs. The distinct properties of this non-biphenyl, non-tetrazole ARB might be relevant in the effort to reduce cardiovascular risk, also beyond its blood pressure lowering capacity.

## INTRODUCTION

The purpose of treating patients with increased cardiovascular risk is to reduce that risk. It is well established that blood pressure lowering per se substantially reduces cardiovascular risk. Both the renin-angiotensin system (RAS) and the sympathetic nervous system (SNS) play an important role in the pathogenesis of various forms of hypertension. Independent of their effect on blood pressure, these systems also contribute to the pathophysiology of both structural and functional cardiovascular abnormalities. As a consequence, counteracting the mechanisms involved in the pathogenesis of cardiovascular organ damage is also important, independent of any direct blood pressure lowering effect.

There is conclusive evidence that the RAS and SNS systems do not operate independently, but that there are multiple interactions on different levels of the cardiovascular system. It is well established that angiotensin converting enzyme (ACE) inhibitors and angiotensin II receptor blockers (ARBs) reduce the effects of angiotensin II Fig. (**1**). These therapies also appear to have sympatholytic properties, which may be particularly important in determining the efficacy of these agents in reducing cardiovascular risk, since there is abundant evidence that sympathetic hyperactivity is associated with poor clinical outcome.

In this brief review, we outline the interactions between the RAS and the SNS. Because eprosartan is a relatively new ARB, chemically distinct from other ARBs, it seems appropriate to summarize available evidence of eprosartan as sympatholytic agent, and briefly review data on experience with this compound in various hypertensive populations.

## ANGIOTENSIN – SYMPATHETIC INTERACTIONS

RAS/SNS interactions appear bi-directional and occur at different sites in the chain of events leading to angiotensin II and noradrenaline release, the two major transmitters of these two systems [1]. There is clear experimental evidence that the sympathetic outflow to the kidneys regulates renin release. Electrical stimulation of the renal nerves, as well as of certain central nervous system areas, causes an increase in renin release. There is a large body of evidence in various experimental settings indicating that angiotensin II facilitates the sympathetic nervous system on different levels. It has been shown that intra-cerebral infusion of angiotensin II causes a pressor response associated with an increase in vascular resistance. On a peripheral level, angiotensin II also elicits stimulatory action, because it stimulates neural transmission across sympathetic ganglia, potentiates at the presynaptic level noradrenaline release from sympathetic nerve terminals and amplifies the alpha-adrenoceptor mediated vasoconstrictor response to endogenous noradrenaline Fig (**2**). Further, angiotensin II exerts inhibitory effects on baroreflex modulation of the heart rate and sympathetic drive. The idea that AngII directly stimulates centrally originated sympathetic outflow in humans was tested in an elegant study. Intravenous infusion of AngII raised blood pressure and suppressed muscle sympathetic nerve activity. But during simultaneously infusion of nitroprusside to control blood pressure rise, MSNA increased. An identical experiment with phenylephrine infusion showed no effect on MSNA [2]. 

Various pathological conditions are associated with hyperactivity of both the RAS and SNS systems, including hypertension, heart failure, kidney disease, hypertension associated with obesity and obstructive sleep apnea syndrome amongst others. In the majority of these conditions both the RAS and the SNS are activated. Because circulating renin is (almost) exclusively produced by the kidneys, it is logical to question how the kidneys might contribute to the increased activities of the two pressor systems in the above-mentioned conditions. The precise role of a local i.e. tissue RAS is much more difficult to quantify and evaluate. 

Experimental studies have shown several pathophysiological mechanisms through which the diseased kidneys can be involved. Inappropriate renin secretion in relation to the state of sodium-volume balance has long been recognized. In humans, intravenous infusion of angiotensin II stimulates central sympathetic outflow.

Renal ischemia can lead to sympathetic activation. During renal ischemia, adenosine is released and adenosine evokes an increase in afferent renal nerve traffic, as can be shown during adenosine infusion in the renal artery of uninephrectomized dogs. Even a small injury in one kidney caused by intrarenal injection of phenol, which does not affect glomerular filtration rate, leads to hypertension in association with an increased central sympathetic activity. In these animal models renal denervation results in a reduction or total prevention of hypertension. Additionally, in the phenol hypertension model nephrectomy of the injured kidney several weeks after the induction of renal damage results in normalisation of blood pressure. Thus kidney injury in experimental conditions can lead to sympathetic hyperactivity and hypertension and this hyperactivity is associated with activation of renal afferent nerves. The signal from the diseased kidneys travels *via* the afferent renal nerves to the central nervous system Fig. (**3**). 

Some forms of essential hypertension, hypertension associated with chronic kidney disease, renovascular hypertension, heart failure, and obesity/metabolic syndrome are all associated with both an activated RAS and an activated SNS. Agents that interfere with the RAS are particularly effective in reducing sympathetic activity when the RAS is activated. The apparent marked parallelism in the activities of these two systems may be interpreted as indicating a cause and effect relation or a common origin, i.e. kidney ischemia Fig. (**3**). Given the importance of sympathetic activity in determining cardiovascular morbidity and mortality, it is important to quantity the ability of agents that affect the RAS to reduce sympathetic activity.

## EPROSARTAN: EFFECTS ON THE RENIN-ANGIOTENSIN AND THE SYMPATHETIC NERVOUS SYSTEM.

Eprosartan is chemically-distinct from the other ARBs in its class [3, 4]. It is the only ARB that belongs to the non-biphenyl, non-tetrazole class of compounds and does not contain a biphenyl, tetrazole moiety Fig. (**4**). The antagonistic properties of eprosartan on the angiotensin II receptor type 1 (AT1) have been extensively documented [5]. Unlike most other ARBs, which show noncompetitive kinetics, eprosartan is a pure competitive antagonist. Based on the absence of a direct comparison of eprosartan with other ARBs, the clinical relevance of the difference in chemical structure and kinetics has to be judged as unclear.

Angiotensin II may stimulate the SNS on various levels Fig (**2**). Consequently, ARBs can block this stimulatory effect of angiotensin II at these different levels. The effects of eprosartan on sympathetic activity have been studied in experimental conditions. Eprosartan induces dual blockade of angiotensin II receptors both pre- and post-synaptically Fig. (**2**). Importantly, it has been shown that eprosartan crosses the blood-brain barrier, which pro-bably contributes to its antihypertensive efficacy [6]. The question whether ARBs have sympatholytic effects is addressed in experimental studies. For instance, Balt *et al.* [7] compared in the pithed rat model the efficacy of valsartan, candesartan, embusartan, telmisartan, eprosartan, losartan and irbesartan on pre- and postjuncional AngII receptors. Eprosartan appeared the most effective agent with respect to prejunctional effects. Ohlstein *et al.* [8] studied activation of sympathetic outflow through spinal cord stimulation in the pithed rat. Eprosartan inhibited sympathetic outflow – but not losartan, valsartan or irbesartan. This difference might mean that eprosartan is a more effective antagonist of prejunctional AngII receptors that augment noradrenaline release. Shetty and Delgrande [9] found that eprosartan inhibited neuronal noradrenaline release in a rat atria model by AngII enhancement of electrical stimulation, which might be interpreted as an effect on prejunctional AngII receptors. Criscione *et al.* [10] found valsartan had no effect on stimulation induced activation of the sympathetic nervous system in pithed rats. Nap and colleagues [11] showed in isolated rabbit thoraric aorta that the AngII enhanced electrical field stimulation evoked sympathetic transmission was more effectively inhibited by candesartan than eprosartan. It is important to emphasize that studies are difficult to compare because protocol, experimental design, dosage and methodology vary substantially between studies. However, they seem to indicate that ARBs have a clear action on both pre- and postjunctional AngII receptors, and that there might be differences in this respect between the various compounds. Importantly, also in humans, there is some indication of different efficacy. For instance, in hypertensive patients Arosio *et al.* [12] reported that 15 days of eprosartan (600 mg od) blocks noradrenergic effects during stress more effectively than valsartan (160 mg od). These data suggest that in hypertensive humans there could be a difference between ARBs in their efficacy to block the noradrenergic system and any such difference might be meaningful [13].

## EPROSARTAN IN CLINICAL PRACTICE

### Essential Hypertension

The clinical efficacy of eprosartan has been established in a number of trials, both against placebo and other compounds. This agent clearly has a 24-hour blood pressure lowering effect during chronic treatment at the standard dose of 600 mg/day [14]. Eprosartan showed similar or greater antihypertensive effect compared with enalapril. For instance, both agents reduced blood pressure, but the response rate at 12 weeks for eprosartan (200-300 mg bid) was greater than with enalapril (5-20 mg od) (70% vs 63%, p<0.05) [15]. In severely hypertensive patients, eprosartan (200-400 mg bid) more effectively reduced systolic blood pressure with no difference in diastolic blood pressure reduction as compared to enalapril (10-40 mg od) after 8 weeks [16]. In a study of mainly elderly patients once daily 600-800 mg eprosartan or once daily enalapril 5-20 mg for 12 weeks reduced blood pressure to a similar extent, with similar proportions of patients in both treatment arms achieving a response [17]. In yet another study in essential hypertensives, once daily eprosartan (400-800 mg) resulted in significantly greater decrease in blood pressure than placebo [18].

A recent study in essential hypertensive patients, assessing both brachial and central blood pressures (pulse wave analysis, Sphygmo Cor System) presented similar reductions during eprosartan (600 mg od) and atenolol (50 mg od) in peripheral blood pressure after 6 weeks. Both agents reduced central systolic pressure, albeit eprosartan more so than atenolol, but only eprosartan reduced wave reflections. Central pressures more closely relate to clinical outcome than peripheral pressures, suggesting an advantage of eprosartan over atenolol [19]. These findings confirm earlier studies indicating differential effects of various antihypertensive agents on central pressures (for instance the CAFE Study [20, 21]) and suggest a mechanism to support the meta-analyses that have challenged the recommendation to use a beta-blocker in uncomplicated hypertension [22-24]. However, superiority can only be really proven by direct comparison in properly designed studies with relevant clinical endpoints.

In patients with mild hypertension and type 2 diabetes once daily eprosartan (600 mg od) and telmisartan (40 mg od) were equally effective in reducing blood pressure during a 12 months study [25]. Obesity is often associated with a raised sympathetic activity leading not only to hypertension, but also insulin resistance [26]. Essential hypertension is a heterogenous condition. Some forms are associated with increased sympathetic activity whilst others are not. Krum *et al.* showed that in mild to moderate essential hypertension ARB treatment (eprosartan 600 mg and losartan 50 mg, both od during 4 weeks) did not affect muscle sympathetic nerve activity, which is the centrally originated postganglionic sympathetic activity [27]. 

### Renal Disease

There is clear evidence that chronic renal disease is often characterised by the presence of sympathetic hyperactivity and that sympathetic activity is associated with poor cardiovascular outcomes in chronic renal failure patients [28-30]. It is important to realize that already minor kidney injury, not necessarily affecting kidney function, may cause sympathetic hyperactivity. This increase in sympathetic activity is thought to both initiate and sustain the elevated blood pressure that contributes to organ damage and adverse cardiovascular events [31]. It may also affect the progression of renal failure [30]. 

Current evidence suggests there is no accumulation of eprosartan at the recommended dose of 600 mg, regardless of renal function. Chronic treatment (> 6 weeks) with enalapril (20 mg od) and losartan (100 mg od) significantly reduced but do not normalized muscle sympathetic nerve activity in patients with hypertensive chronic renal failure [32, 33]. Eprosartan (600 mg od) reduced muscle sympathetic nerve activity, blood pressure and also heart rate (all p<0.05), suggesting that not only the sympathetic activity towards the resistance vasculature but also the cardiac sympathetic activity is decreased with eprosartan. Combining eprosartan with the centrally-acting sympatholytic agent moxonidine normalised both blood pressure and muscle sympathetic nerve activity in hypertensive normovolemic patients with chronic renal failure [32]. Studies directly comparing effects on muscle sympathetic nerve activity, which is considered one of the gold standards for quantifying sympathetic activity in humans, of eprosartan and other agents in disease states with high sympathetic activity are presently not available.

Experimental studies with eprosartan suggest that it might have a benefit in the prevention or delay of renal damage in hypertensive patients with renal impairment [34, 35]. Frank *et al.* [36] showed that eprosartan (600 mg od for 7 days) preserves renal circulation during states of neurohumoral activation, suggesting an important renoprotective effect of this compound. Osei *et al.* [37] noted an enhancement of the renal vasodilator effect of eprosartan (600 mg) during hyperglycemia consistent with activation of the intrarenal RAS.

### The Elderly

Isolated systolic hypertension is the most common form of hypertension in the elderly [38, 39]. Eprosartan has been shown to be an effective agent in the elderly. In a double-blind study versus placebo in 283 elderly patients with a mean age of 70 years eprosartan once-daily at doses of 600-1200mg during 3 months produced a significant reduction in systolic blood pressure (eprosartan vs. placebo: 16 and 8 mmHg, p<0.0001) [40]. For patients who did not respond to eprosartan alone, the addition of hydrochlorothiazide provided additive reduction in systolic blood pressure (eprosartan vs. placebo: 22 and 14 mmHg, p<0.002). In a 26-week comparative study with enalapril (5 – 20 mg od), eprosartan at doses of 200-300mg twice daily, alone or in combination with hydrochlorothiazide once daily, was shown to be safe and effective in hypertensive patients over 65 years of age [41]. Antihypertensive efficacy of eprosartan and enalapril did not differ. In an equal percentage of patients, the diuretic was added.

In the ETAPA (Estudio de la Efectividad del Tratamiento antihipertensivo sobre la Presion de Pulso en adultos) study from Spain involving approximately 4,000 hypertensive patients (mean age 67 y), eprosartan (600 mg) alone (87%) or in combination with a diuretic decreased systolic, diastolic and mean (resp. 26 mmHg, 13 mmHg and 17 mmHg, all p<0.01) blood pressure in hypertensive patients and was associated with a reduction in pulse pressure / mean blood pressure ratio (62 => 58%, p<0.05) [42]. Also in the Robles *et al.* study, eprosartan (600 mg od for 16 weeks) in the primary care setting was well tolerated and effective in over 600 patients (mean age 63 y) in reducing blood pressure (systolic, diastolic and mean blood pressure resp: 26, 13 and 17 mmHg, all p<0.001) as well as pulse pressure (13 mmHg, p<0.001). Also the pulse pressure / mean arterial pressure ratio decreased (62 => 59%, p< 0.001) [43].

### Other High Risk Populations

The MOSES study (MOrbidity and mortality after Stroke, Eprosartan compared with nitrendipe for Secondary prevention) is a large, prospective, randomised outcome study comparing the addition of 600 mg eprosartan or 10 mg nitrendipine to existing therapy in patients with a history of proven stroke or transient ischaemic attack [44]. Background therapy did not differ between the two groups. In MOSES, 1405 patients were followed up for 2.5 years with eprosartan or nitrendipine. The primary outcomes were total mortality and cardiovascular and cerebrovascular disease and the secondary outcomes were mental status, neurological status and ambulatory blood pressure. Blood pressure did not differ between the treatment arms. There was a significant reduction of 21% in the primary endpoint (all-cause mortality and the number of cardiovascular and cerebrovascular events, including all recurrent events) and of 25% (p=0.026) in the recurrence of stroke, and of 30% in first time cardiovascular events. This important study indicates that meaningful prevention, beyond an effect on blood pressure, can be obtained in a high risk population. Although there has been some concern on the reliability and relevance of the study [45], it extends the existing information on the effects of ARBs in high risk patients. Some, but not all, other studies with ARBs suggest that these agents especially reduce strokes. For instance in the LIFE study in hypertensive patients with left ventricular hypertrophy losartan based therapy reduced stroke better than beta-blocker based therapy [46]. In contrast, in the VALUE study, valsartan was nonsignificantly worse than amlodipine in stroke prevention, but post hoc analyses suggested that this may have been attributable to less effective blood pressure control during valsartan during the first 6 months [47]. In ACCESS, candesartan (or placebo) was given after 7 days after onset of stroke. Neurological outcome after 1 year in the ARB treated group was better than in the placebo group [48]. The mechanism of this possible beneficial effect may lie in the selective blocking by ARBs of the deleterious effects mediated through the AT1 receptor, whereas the effects on the AT2 receptor are unaffected or even enhanced [49]. AT2 receptor seem to mediate beneficial effects on endothelium through decreased coagulation and inflammation and these receptors also protect brain tissue from ischemia in experimental models [50, 51].

### Ongoing Studies

Although different in terms of structure and mode of action, eprosartan has, to date, been seen as one of many sartans. Ongoing studies are now focusing in other areas where reduction of sympathetic activity might be expected to show benefit. 

The STARLET study (Stress-induced Hypertension at the Workplace) will examine the prevalence of ‘job-strain’ hypertension and cardiovascular outcomes. Job-strain hypertension is defined as a difference of at least 5mm diastolic blood pressure and/or 8mm Hg systolic blood pressure between work and weekend mean daytime ambulatory blood pressure. First results show that eprosartan effectively reduced blood pressure [51].

The OSCAR study will be a long-term, open-label, study examining the effects of eprosartan-based therapy (600 mg od) on systolic blood pressure and cognitive function. Results from this study are also awaited with interest [52, 53]. 

## CONCLUSION

The goal of antihypertensive treatment is not only to lower blood pressure but also to reduce risk of cardiovascular morbidity and mortality. It is important not only to quantify the blood pressure lowering effect of antihypertensive compounds, but also to assess their ability to counteract mechanisms involved in the pathogenesis of cardiovascular morbidity and mortality, such as the hyperactivities of the renin-angiotensin system and the sympathetic nervous system. Eprosartan is an effective antihypertensive agent and clearly belongs to the class of ARBs, reversing effects of the renin and the sympathetic system. Experimental and pharmacokinetic studies have shown differences with the other ARBs. Since direct comparison of effects of eprosartan and other ARBs on meaningful clinical endpoints is not available, the clinical relevance of these differences has to be judged as unclear.

## Figures and Tables

**Fig. (1).**
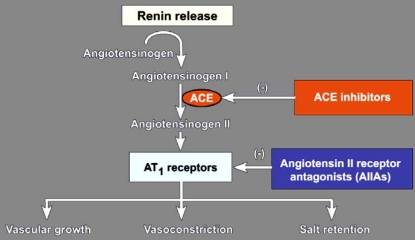
Schematic representation of the renin-angiotensin system and the sites of action of angiotensin converting enzyme (ACE) inhibitors and angiotensin II receptor blockers.

**Fig. (2).**
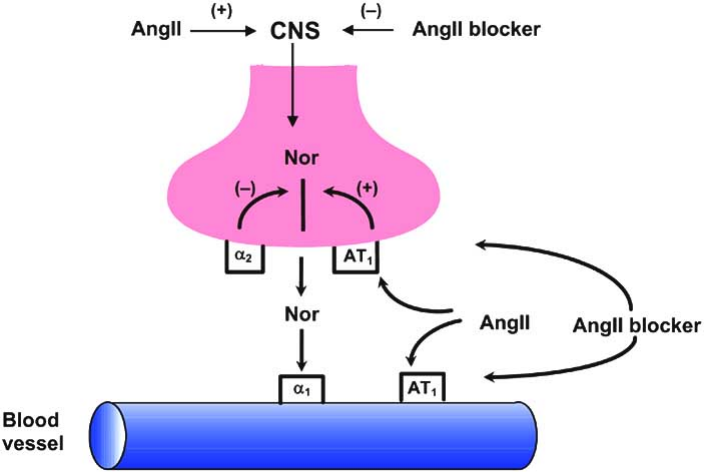
Schematic representation of the central nervous system (CNS) and a neuro-effector junction. Levels are indicated where angiotensin II (Ang II) can enhance (+) sympathetic activity and where angiotensin II receptor blocker (Ang II blocker) can reduce (-) sympathetic activity. Nor = noradrenaline, α = alpha-adrenoceptor, AT = angiotensin receptor

**Fig. (3).**
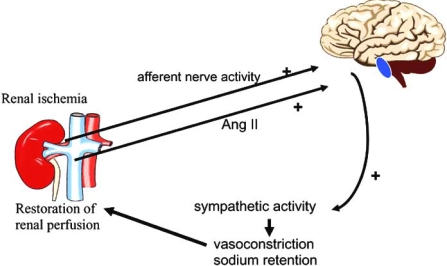
Schematic representation of the kidney involvement in the pathogenesis of sympathetic hyperactivity. Already minimal kidney damage, not necessarily affecting kidney function, results in area(s) of ischemia. Increased plasma levels of angiotensin II and/or increased afferent renal nerve activity stimulates the central nervous system to increase central sympathetic outflow, which results in sodium retention and vasoconstriction which are meant to restore kidney perfusion.

**Fig. (4).**
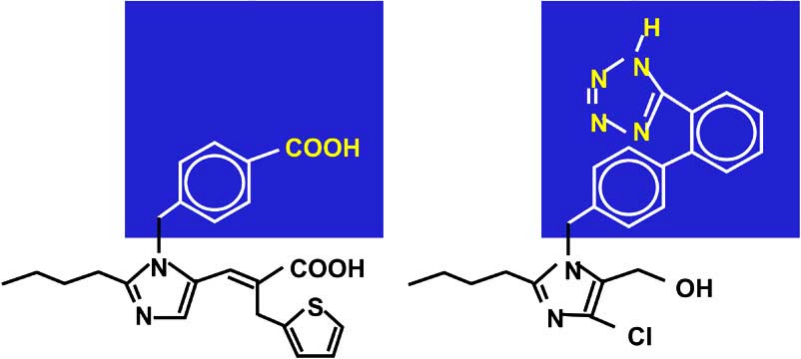
Eprosartan (left) is a non-biphenyl, non-tetrazole angiotensin II receptor blocker (ARB), other ARBs are biphenyl tetrazoles (right).
